# Bioactive 2(1*H*)-Pyrazinones and Diketopiperazine Alkaloids from a Tunicate-Derived Actinomycete *Streptomyces* sp.

**DOI:** 10.3390/molecules21091116

**Published:** 2016-08-24

**Authors:** Lamiaa A. Shaala, Diaa T. A. Youssef, Jihan M. Badr, Steve M. Harakeh

**Affiliations:** 1 Natural Products Unit, King Fahd Medical Research Center, King Abdulaziz University, Jeddah 21589, Saudi Arabia; lshalla@kau.edu.sa; 2 Suez Canal University Hospital, Suez Canal University, Ismailia 41522, Egypt; 3 Department of Natural Products, Faculty of Pharmacy, King Abdulaziz University, Jeddah 21589, Saudi Arabia; jibrahim@kau.edu.sa; 4 Department of Pharmacognosy, Faculty of Pharmacy, Suez Canal University, Ismailia 41522, Egypt; 5 Special Infectious Agents Unit, Fahd Medical Research Center, King Abdulaziz University, Jeddah 21589, Saudi Arabia; sharakeh@gmail.com

**Keywords:** Red Sea *Didemnum* sp., *Streptomyces* sp. Did-27, alkylated 2(1*H*)-pyrazinone derivatives, diketopiperazine alkaloids, cancer cell lines, antiproliferative and cytotoxic activities

## Abstract

As a part of our ongoing effort to allocate marine microbial bioactive leads, a tunicate-derived actinomycete, *Streptomyces* sp. Did-27, was investigated. Three new 2(1*H*)-pyrazinones derivatives, (*S*)-6-(*sec*-butyl)-3-isopropylpyrazin-2(1*H*)-one (**1**), (*S*)-3-(*sec*-butyl)-6-isopropylpyrazin-2(1*H*)-one (**2**) and (*S*)-6-(*sec*-butyl)-3-isobutylpyrazin-2(1*H*)-one (**3**), together with the known (1*H*)-pyrazinones analogues deoxymutaaspergillic acid (**4**), 3,6-diisobutyl-2(1*H*)-pyrazinone (**5**) and 3,6-di-*sec*-butyl-2(1*H*)-pyrazinone (**6**), and the diketopiperazine alkaloids cyclo(6-OH-d-Pro-l-Phe) (**7**), bacillusamide B (**8**), cyclo(l-Pro-l-Leu) and cyclo(l-Pro-l-Ile) (**10**) were isolated from this strain. The structures of the compounds were determined by study of their one- and two-dimensional NMR spectra as well as high-resolution mass spectral determinations. Compound **4** was reported previously as a synthetic product, while compound **6** was reported as 2-hydroxy-3,6-di-*sec*-butylpyrazine. Herein, we report the complete NMR data for compounds **4** and **6**. The compounds were evaluated for their cytotoxic activities against three cell lines. Compound **5** showed potent and selective activity against HCT-116 cell line with IC_50_ of 1.5 μg/mL, while **1**–**10** showed variable cytotoxic activities against these cancer cell lines. These results provide further understanding about the chemistry and bioactivities of the alkylated 2(1*H*)-pyrazinone derivatives.

## 1. Introduction

The genus *Streptomyces* was first described by Waksman and Henrici [1] and is considered as a promising resource for bioactive natural products and drug discovery [2,3]. More than 75% of the important drugs are produced by members of the *Streptomyces* [4] including a wide array of antibiotics and anticancer drugs [5,6]. As a part of our ongoing effort to allocate bioactive leads from marine microbes [7,8,9], we have investigated a tunicate-derived actinomycete, *Streptomyces* sp. Did-27. Bioassay-guided fractionation of the active fractions of an organic extract of this strain resulted in the isolation and identification of three new alkylated 2(1*H*)-pyrazinone derivatives including (*S*)-6-(*sec*-butyl)-3-isopropylpyrazin-2(1*H*)-one (**1**), (*S*)-3-(*sec*-butyl)-6-isopropylpyrazin- 2(1*H*)-one (**2**) and (*S*)-6-(*sec*-butyl)-3-isobutylpyrazin-2(1*H*)-one (**3**), together with deoxymutaaspergillic acid (**4**) [10,11,12,13,14,15,16], 3,6-diisobutyl-2(1*H*)-pyrazinone (**5**) [15,16,17,18,19,20,21,22,23] and 3,6-di-*sec*-butyl-2(1*H*)-pyrazinone (**6**). Compound **6** was published before as 2-hydroxy-3,6-di-*sec*-butylpyrazine [23,24,25,26]. In addition, four diketopiperazine alkaloids including cyclo(6-OH-d-Pro-l-Phe) (**7**) [27], bacillusamide B (**8**) [28], cyclo(l-Pro-l-Leu) (**9**) [29] and cyclo(l-Pro-l-Ile) (**10**) [30] were isolated from the extract of the marine *Streptomyces* sp. The structures of the compounds were determined by extensive interpretation of their spectral data including 1D and 2D NMR and HRMS. Due to the lack or incomplete NMR data of compounds **4** and **6** in the literature, the complete NMR data of these compounds were presented. The isolated compounds were evaluated for their cytotoxic activity against colorectal carcinoma, hepatocellular carcinoma and breast cancer cell lines. Compound **5** showed potent and selective activity against HCT-116 cell line with IC_50_ of 1.5 μg/mL, while **1**–**10** showed variable cytotoxic activities against these cancer cell lines. These results provide further and deeper insight into the chemical diversity and biological activities the alkylated 2(1*H*)-pyrazinone derivatives.

## 2. Results and Discussion

**Figure 1:**
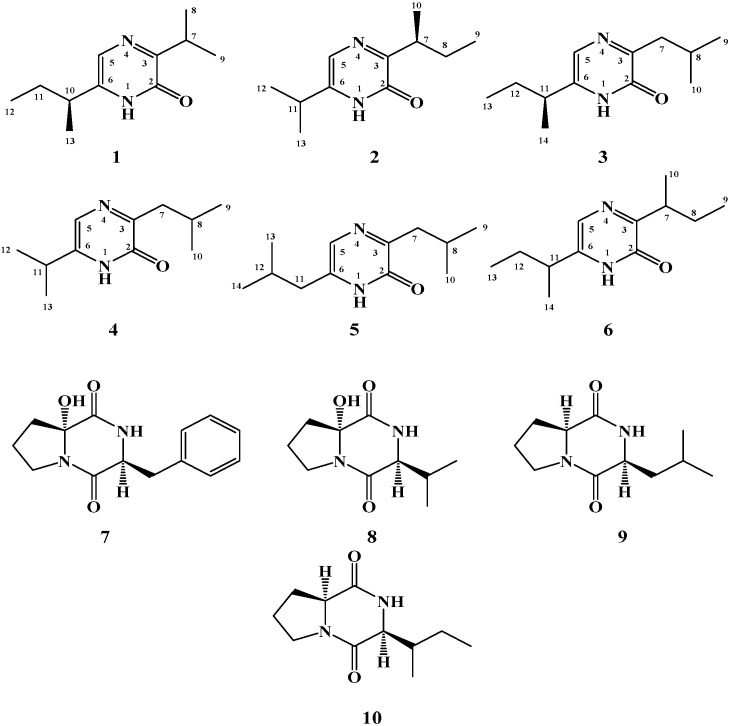
Structures of compounds **1**–**10**.

**Figure 2:**
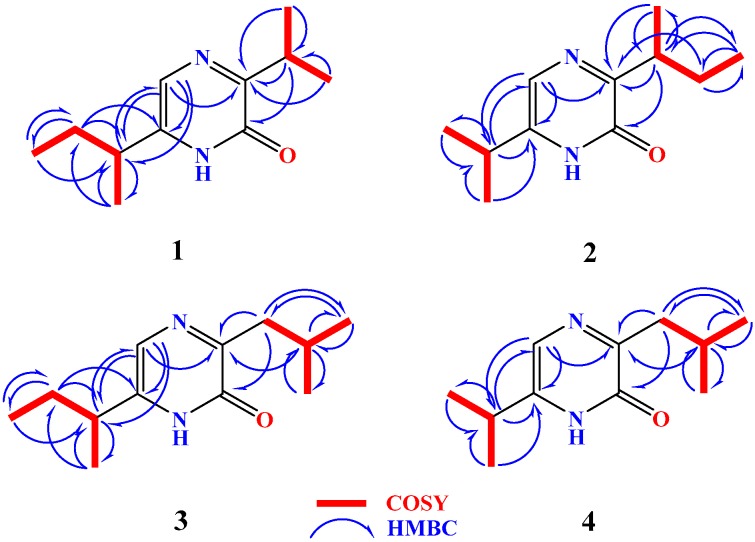
Key COSY and HMBC correlations of **1**–**4**.

Compound **1** (Figure 1) possesses a molecular formula C_11_H_18_N_2_O as deduced from the HRESIMS pseudomolecular ion peak at *m/z* 195.1499 [M + H]^+^, requiring four degrees of unsaturation. The IR spectrum showed characteristic bands for an amidic carbonyl (1643 cm^−1^) and an amino group (3430 cm^−1^). The ^1^H and ^13^C NMR spectra of **1** together with the HSQC experiment displayed signals characteristic for a 3,6-disubstituted-2(1*H*)-pyrazinone skeleton [10,17]. This was evident from the ^1^H/^13^C signals at δ_H_ 11.19 (1H, s, NH), δ_C_ 156.9 (qC, C-2), δ_C_ 161.8 (qC, C-3), δ_H_/δ_C_ 7.17 (1H, s, H-5)/120.9 (CH, C-5) and δ_C_ 141.6 (qC, C-6) (Table 1). In the COSY spectrum, two spin-spin coupling systems for isopropyl and *sec*-butyl subunits could be traced within **1**. The signals at δ_H_/δ_C_ 3.40 (1H, sept, *J* = 6.6 Hz, H-7)/30.1 (CH, C-7), 1.24 (3H, d, *J* = 6.6 Hz, H_3_-8)/19.9 (CH_3_, C-8) and 1.25 (3H, d, *J* = 6.6 Hz, H_3_-9)/20.0 (CH_3_, C-9) were assigned as an isopropyl group. While the presence of a *sec*-butyl group in **1** was supported by the signals at 2.51 (1H, sixth, *J* = 7.2 Hz, H-10)/37.1 (CH, C-10), 1.70 (1H, m, H-11a), 1.62 (1H, m, H-11b)/28.5 (CH_2_, C-11), 0.90 (3H, t, *J* = 7.2 Hz, H_3_-12)/11.8 (CH_3_, C-12) and 1.30 (3H, d, *J* = 6.6 Hz, H_3_-13)/18.8 (CH_3_, C-13). The placement of the isopropyl and *sec*-butyl subunits at C-3 and C-6, respectively, was supported by HMBC correlations of H-7/C-2, H-7/C-3, H_3_-8/C-3, H_3_-9/C-3, H-5/C-6, H-5/C-10, H-10/C-6, H-10/C-5, H_3_-13/C-6 (Figure 2). Additional HMBC correlation within the two alkyl moieties were shown in Figure 2. The configuration at C-10 in **1** was proposed to be 10*S* as established from the positive sign of the optical rotation of +12.5° (compared to +11.3° for the synthetic compound (*S*)-6-(*sec*-butyl)-3-isbutylpyrazin-2(1*H*)-one [31]. Thus, compound **1** was assigned as (*S*)-6-(*sec*-butyl)-3-isopropylpyrazin-2(1*H*)-one and is considered as a new natural compound.

Compound **2** (Figure 1) showed a molecular formula C_11_H_18_N_2_O as established from the HRESIMS pseudomolecular ion peak at *m/z* 195.1499 [M + H]^+^, requiring four degrees of unsaturation. The IR displayed bands for an amidic carbonyl (1640 cm^−1^) and an amino group (3435 cm^−1^). The ^1^H and ^13^C NMR spectra of **1** together with the HSQC experiment displayed signals characteristic for a 3,6-disubstituted-2(1*H*)-pyrazinone skeleton (Table 1). Investigation of the ^1^H and ^13^C NMR spectra, ^1^H-^1^H COSY and HSQC experiments of **2** supported the presence of three subunits including 3,6-disubstituted-2(1*H*)-pyrazinone, isopropyl and *sec*-butyl moieties as observed in **1**. Since compounds **1** and **2** possess the same molecular formula, same number of degrees of unsaturation as well as same subunits, the difference between both compounds was in the placement of the alkyl subunits. In compound **2**, the isopropyl and *sec-*butyl subunits exist at C-6 and C-3, respectively, instead of C-3 and C-6 in **1**. These placements were unambiguously supported by HMBC correlations of H-11/C-5, H-11/C-6, H-5/C-11 as well as HMBC cross-peaks of H-7/C-2, H-7/C-3 and H_3_-10/C-3 (Figure 2). Additional HMBC correlation unambiguously supported the assignment of all carbon signals of **2** (Figure 2). Again, the configuration at C-7 in **2** was assigned as 7*S* based on the negative sign of the optical rotation. Compound **2** displayed a negative optical rotation of −3.4° (compared to +1.99° for paenibacillin A) [32]. Thus, compound **2** was considered as a new compound and was assigned (*S*)-3-(*sec*-butyl)-6-isopropylpyrazin-2(1*H*)-one.

Compound **3** (Figure 1) with a molecular formula C_12_H_20_N_2_O as established from the HRESIMS pseudomolecular ion peak at *m/z* 209.1655 [M + H]^+^. Compound **3** is 14 mass unit larger than **1** suggesting the presence of additional methylene group in **3**. Its UV spectrum displayed absorption maxima at 325 and 227 nm. The IR spectrum showed absorption bands for an amidic carbonyl (1645 cm^−1^) and an amino group (3440 cm^−1^). Again, the ^1^H and ^13^C NMR spectra of **3** together with the HSQC experiment displayed signals characteristic for a 3,6-disubstituted-2(1*H*)-pyrazinone skeleton (Table 2). Study of the ^1^H and ^13^C NMR spectra, ^1^H-^1^H COSY and HSQC experiments of **3** supported the presence of three subunits including 3,6-disubstituted-2(1*H*)-pyrazinone, isobutyl and *sec*-butyl subunits. The placement of the isobutyl and *sec*-butyl subunits at C-3 and C-6 was unambiguously supported by HMBC correlations of H_2_-7/C-2, H_2_-7/C-3 and H-8/C-3, as well as HMBC cross-peaks of H-11/C-5, H-11/C-6, H_2_-12/C-6, H_3_-14/C-6 and H-5/C-11 (Figure 2). Additional HMBC correlations unambiguously supported the assignment of all carbon signals of **3** (Figure 2). Again, the configuration at C-11 in **3** was proposed to be 11*S* based on the positive sign of the optical rotation. Compound **3** displayed a positive optical rotation of +11.5° (compared to +11.3° for (*S*)-6-(*sec*-butyl)-3-isobutylpyrazin-2(1*H*)-one [31]. Compound **3** was reported before as a synthetic product [18], but this is the first report of this compound from a natural source. Accordingly, compound **3** is reported here as a new natural product and was assigned as (*S*)-6-(*sec*-butyl)-3-isobutylpyrazin-2(1*H*)-one.

Compound **4** (Figure 1) showed a molecular formula C_11_H_18_N_2_O as established from the HRESIMS pseudomolecular ion peak at *m/z* 195.1497 [M + H]^+^. It possesses the same molecular formula of **1**. The ^1^H and ^13^C NMR spectra of **4** together with the HSQC experiment supported the presence of a 3,6-disubstituted-2(1*H*)-pyrazinone moiety (Table 2). Study of the ^1^H and ^13^C NMR spectra, ^1^H-^1^H COSY and HSQC experiments of **4** supported the presence of three subunits including 3,6-disubstituted-2(1*H*)-pyrazinone, isopropyl and isobutyl subunits. The placement of the isobutyl and isopropyl subunits at C-3 and C-6 was unambiguously supported by HMBC correlations (Figure 2). Therefore, compound **4** was assigned as deoxymutaaspergillic acid [10,11,12,13,14,15,16].

Compound **5** (Figure 1) with a molecular formula C_12_H_20_N_2_O as established by HRESIMS. It was identified as 3,6-diisobutyl-2(1*H*)-pyrazinone as established by study of its NMR data (Table 3) as well as by comparison with the literature [15,16,17,18,19,20,21,22,23]. Compound **6** (Figure 1) possesses a molecular formula of C_12_H_20_N_2_O as established by HRESIMS. Study of the ^1^H and ^13^C NMR spectra, ^1^H-^1^H COSY and HSQC experiments of **3** supported the presence of three subunits including 3,6-disubstituted-2(1*H*)-pyrazinone and two *sec*-butyl subunits (Table 3). Therefore, it was identified as 3,6-di-*sec*-butyl-2(1*H*)-pyrazinone as established by study of its NMR data (Table 3).

Compound **6** was reported before as 2-hydroxy-3,6-di-*sec*-butylpyrazine and was identified by mass spectroscopy only [23,24,25,26]. To the best of our knowledge, there is no available complete NMR data for compound **6**. Thus, **6** was assigned as 3,6-di-*sec*-butyl-2(1*H*)-pyrazinone and its complete NMR data are presented in Table 3.

The diketopiperazine alkaloids **7–10** were identified by extensive study of their spectral data including HRESIMS, 1D (^1^H and ^13^C) and 2D (COSY, HSQC and HMBC) NMR data as well as by comparison with the literature. Thus, the compounds were identified as cyclo(6-OH-d-Pro-l-Phe) (**7**) [27], bacillusamide B (**8**) [28], cyclo(l-Pro-l-Leu) (**9**) [29] and cyclo(l-Pro-l-Ile) (**10**) [1].

Compounds **1**–**10** were evaluated for their antiproliferative and cytotoxic activities in the sulforhodamine B (SRB) assay against HCT-116 (colorectal carcinoma, ATCC CCL-247), HepG2 (hepatocellular carcinoma, ATCC HB-8065) and MCF-7 (breast cancer, ATCC HTB-22). Compound **5** showed potent and selective activity agaisnt HCT-116 cell line with IC_50_ of 1.5 µg/mL, while all other compounds were moderately active against this cell line with IC_50_ of 16–35 µg/mL. Similarly, all compounds were moderately active against MCF-7 with IC_50_ of 10–35 µg/mL (Table 4). Finally, all compounds were weakly active against HepG2 with IC_50_ ≥ 50 µg/mL when tested against HepG2 cell line. The results of the antiproliferative and cytotoxic activities of **1**–**10** are displayed in Table 4.

## 3. Materials and Methods

### 3.1 Experimental

#### General Experimental Procedures

Optical rotations were measured on a JASCO DIP-370 digital polarimeter at 25 °C at the sodium D line (589 nm). UV spectrum were recorded on a Hitachi 300 spectrometer. IR spectra were measured on a Shimadzu Infrared-400 spectrophotometer (Shimadzu, Kyoto, Japan). 1D and 2D NMR spectra (chemical shifts in ppm, coupling constants in Hz) were recorded on Bruker Avance DRX 600 MHz spectrometers (Bruker, Rheinstetten, Germany) using CDCl_3_ and CD_3_OD as solvents. NMR spectra were referenced to the residual protonated solvent signals (CHCl_3_: 7.26 ppm for ^1^H and 77.0 ppm for ^13^C; CH_3_OD: 3.30 ppm for ^1^H and 49.0 ppm for ^13^C). Positive ion HRESIMS data were obtained with a Micromass Q-ToF equipped with leucine enkaphalin lockspray, using *m*/*z* 556.2771 [M + H]^+^ as a reference mass. For column chromatography, silica gel (Merck, 70–230 mesh ASTM, Sigma-Aldrich, Darmstadt, Germany) and Sephadex LH-20 (0.25–0.1 mm, Pharmacia, Piscataway, NJ, USA) were used. Precoated silica gel 60 F-254 plates (Merck) were used for TLC. HPLC purifications were performed on a semi-preparative HPLC column (RP18, 5 μm, ARII Cosmosil, 250 × 10 mm, Waters, Nacalai Inc., San Diego, CA, USA).

### 3.2. Biological Materials

#### 3.2.1. The Host Material, *Didemnum* sp.

The marine tunicate *Didemnum* sp. was collected in November 2013 by hands using SCUBA at depths between 15 and 20 m near Obhur, Saudi Arabia. The tunicate material was identified by Dr. Francoise Monniot at Muséum National d’Histoire Naturelle (MNHN), Paris. A voucher specimen was deposited in the MNHN, Paris, under the Registration Number A2-Did c-476.

#### 3.2.2. Actinomycete Material

The actinomycete strain was identified as a member of the genus *Streptomyces* on the basis of 16S rRNA gene sequence analysis. Genomic DNA isolation, PCR amplification of 16S rRNA gene and sequence alignment of the strain were performed as described previously [33]. Its 16S rRNA gene sequence showed 98% similarity with type strains of *Streptomyces flocculus* (DQ442498) and *Streptomyces rangoonensis* (NR_041110).

### 3.3. Fermentation and Extraction

The spores of *Streptomyces* sp. Did-27 were directly cultured in 2000 mL Erlenmeyer flasks containing 500 mL of ISP-2 (ISP2, medium 2 of the International *Streptomyces* Project) [34] fermentation media consisted of yeast extract 4.0 g, malt extract 10.0 g and dextrose 4.0 g and 3.3% sea salt in 1 L distilled water (pH 7.2). The cultures were incubated on a rotatory shaker at 180 rpm at 28 °C for eight days. The whole fermentation broth (20 L) was extracted three times with EtOAc three times. The combined EtOAc solutions were combined and evaporated under reduced pressure to give a dark brown gum (4.3 g).

### 3.4. Isolation and Purification of Compounds ***1**–**10***

The EtOAc extract (4.3 g) was subjected to SiO_2_ VLC eluting with *n*-hexane/CH_2_Cl_2_/MeOH gradients to give six fractions (A–F). Fraction B (390 mg) was subjected to gel filtration on Sephadex LH-20 using MeOH as eluent to give five subfractions (B1–B5). Fraction B3 (139 mg) was further subjected to C18 HPLC separation eluting with 30% ACN to yield **1** (4.5 mg), **2** (1.6 mg), and **3** (3.9 mg). Fraction B4 (180 mg) was subjected to C18 HPLC separation eluting with 35% ACN to yield **7** (6.5 mg), **8** (5.3 mg), **9** (10 mg) and **10** (4.8 mg). Fraction E (320 mg) was purified by gel filtration over Sephadex LH-20 using MeOH giving four subfractions (E1–E4). Fraction E2 (130 mg) was purified by C18 HPLC eluting with 35% ACN to yield **4** (2.9 mg), **5** (4.9 mg) and **6** (4.6 mg).

### 3.5. Spectral Data of the Compounds

Compound **1**: White solid; ${\left[\alpha \right]}_{D}^{25}$+12.5 (*c* 0.1, CHCl_3_); UV (MeOH) *λ*_max_ (log *ε*): 329 (3.65), 227 (3.50) nm; IR (film) *ν*_max_ 3430, 1643 cm^−1^; NMR data: Table 1; HRESIMS *m*/*z* 195.1499 (calcd for C_11_H_19_N_2_O, [M + H]^+^, 195.1497).

Compound **2**: White solid; ${\left[\alpha \right]}_{D}^{25}$−3.4 (*c* 0.1, CHCl_3_); UV (MeOH) *λ*_max_ (log *ε*): 328 (3.65), 227 (3.52) nm; IR (film) *ν*_max_ 3435, 1640 cm^−1^; NMR data: Table 1; HRESIMS *m*/*z* 195.1499 (calcd for C_11_H_19_N_2_O, [M + H]^+^, 195.1497). 

Compound **3**: White solid; ${\left[\alpha \right]}_{D}^{25}$+11.5 (*c* 0.1, CHCl_3_), UV (MeOH) *λ*_max_ (log *ε*): 325 (3.55), 227 (3.50) nm; IR (film) *ν*_max_ 3440, 1645 cm^−1^; NMR data: Table 2; HRESIMS *m*/*z* 209.1655 (calcd for C_12_H_21_N_2_O, [M + H]^+^, 209.1654). 

Compound **4**: White solid; UV (MeOH) *λ*_max_ (log *ε*): 325 (3.53), 227 (3.50) nm; IR (film) *ν*_max_ 3440, 1645 cm^−1^; NMR data: Table 2; HRESIMS *m*/*z* 195.1497 (calcd for C_11_H_19_N_2_O, [M + H]^+^, 195.1497).

### 3.6. Evaluation of Antiproliferative and Cytotoxic Activities of the Compounds

The in vitro antiproliferative and cytotoxic activities of the compounds was evaluated against three human tumor cells including HCT-116 (colorectal carcinoma, CCL-247, ATCC, Manassas, VA, USA), HepG2 (hepatocellular carcinoma, HB-8065, ATCC, Manassas, VA, USA) and MCF-7 (breast cancer, HTB-22, ATCC, Manassas, VA, USA). The effect of compounds **1-10** on cell proliferation and cytotoxicity were evaluated using the sulforhodamine B (SRB) assay as described previously [35]. Doxorubicin were used as positive control drug. The results of the cytotoxic and antiproliferative activities of **1**–**10** are displayed in Table 4.

## 4. Conclusions

In conclusion, investigation of a tunicate-derived actinomycete, *Streptomyces* sp. Did-27, afforded three new compounds, namely (*S*)-6-(*sec*-butyl)-3-isopropylpyrazin-2(1*H*)-one (**1**), (*S*)-3-(*sec*-butyl)-6-isopropylpyrazin-2(1*H*)-one (**2**) and (*S*)-6-(*sec*-butyl)-3-isobutylpyrazin-2(1*H*)-one (**3**) and six previously reported ones including deoxymutaaspergillic acid (**4**), 3,6-diisobutyl-2(1*H*)-pyrazinone (**5**), 3,6-di-*sec*-butyl-2(1*H*)-pyrazinone (**6**), cyclo(6-OH-d-Pro-l-Phe) (**7**), bacillusamide B (**8**), cyclo(l-Pro-l-Leu) and cyclo(l-Pro-l-Ile) (**10**). Their structures were assigned by interpretation of their spectral data. In addition, the complete NMR data for compounds **4** and **6** were reported here for the first time. Compound **5** showed selective and potent active agaisnt colorectal carcinoma cell line (HCT-116) with with IC_50_ of 1.5 µg/mL. All other compounds were moderatly active against MCF-7 and weakly active against HepG2 cell line.

## Figures and Tables

**Table 1:** NMR data of compounds **1** and **2** (600 and 150 MHz, CDCl_3_).

No.	1		2	
	δ_C_ (mult.)	δ_H_ (mult., *J* (Hz))	δ_C_ (mult.)	δ_H_ (mult., *J* (Hz))
1		11.19 (s)		11.28 (s)
2	156.9, qC		157.2, qC	
3	161.8, qC		161.4, qC	
5	120.9, CH	7.17 (s)	120.0, CH	7.21 (s)
6	141.6, qC		142.6, qC	
7	30.1, CH	2.30 (m), 2.15 (m)	36.6, CH	3.23 (sixth, 7.2)
8	20.0, CH_3_	1.25 (d, 6.6)	27.5, CH_2_	1.82 (m), 1.54 (m)
9	19.9, CH_3_	1.24 (d, 6.6)	19.0, CH_3_	0.90 (t, 6.6)
10	37.1, CH	2.51 (sixth, 7.2)	17.7, CH_3_	1.20 (d, 6.6)
11	28.5, CH_2_	1.70 (m), 1.62 (m)	30.0, CH	2.80 (sept, 7.2)
12	11.8, CH_3_	0.90 (t, 7.2)	21.0, CH_3_	1.31 (d, 6.6)
13	18.8, CH_3_	1.30 (d, 6.6)	21.0, CH_3_	1.31 (d, 6.6)

**Table 2:** NMR data of compounds **3** and **4** (600 and 150 MHz, CDCl_3_).

No.	3		4	
	δ_C_ (mult.)	δ_H_ (mult., *J* (Hz))	δ_C_ (mult.)	δ_H_ (mult., *J* (Hz))
1		11.28 (s)		12.06 (s)
2	158.2, qC		157.9, qC	
3	157.1, qC		157.3, qC	
5	121.2, CH	7.18 (s)	120.1, CH	7.19 (s)
6	142.3, qC		143.2, qC	
7	41.6, CH_2_	2.66 (dd, 13.8, 7.2) 2.64 (dd, 13.8, 7.2)	41.5, CH_2_	2.65 (d, 7.2)
8	26.9, CH	2.21 (nonet, 7.2)	26.9, CH	2.21 (nonet, 7.2)
9	22.6, CH_3_	0.96 (d, 6.6)	22.6, CH_3_	0.97 (d, 7.2)
10	22.6, CH_3_	0.96 (d, 6.6)	22.6, CH_3_	0.97 (d, 7.2)
11	37.2, CH	2.54 (sixth, 7.2)	30.0, CH	2.80 (sept, 7.2)
12	28.4, CH_2_	1.74 (m), 1.65 (m)	21.0, CH_3_	1.32 (d, 7.2)
13	11.8, CH_3_	0.90 (t, 7.2)	21.0, CH_3_	1.32 (d, 7.2)
14	18.7, CH_3_	1.31 (d, 6.2)		

**Table 3:** NMR data of compounds **5** and **6** (600 and 150 MHz, CDCl_3_).

No.	5		6	
	δ_C_ (mult.)	δ_H_ (mult., *J* (Hz))	δ_C_ (mult.)	δ_H_ (mult., *J* (Hz))
1		12.05 (s)		11.80 (s)
2	158.0, qC		157.5, qC	
3	157.0, qC		161.2, qC	
5	122.8, CH	7.15 (s)	121.2, CH	7.19 (s)
6	137.3, qC		141.7, qC	
7	41.7, CH_2_	2.65 (d, 7.2)	36.7, CH	3.23 (sixth, 6.6)
8	26.9, CH	2.21 (nonet, 7.2)	28.4, CH_2_	1.72 (m), 1.63 (m)
9	22.6, CH_3_	0.96 (d, 7.2)	12.0, CH_3_	0.90 (t, 7.2)
10	22.6, CH_3_	0.96 (d, 7.2)	18.3, CH_3_	1.31 (d, 7.2)
11	39.5, CH_2_	2.36 (d, 7.2)	37.2, CH	2.53 (sixth, 7.2)
12	28.1, CH	2.03 (nonet, 7.2)	27.5, CH_2_	1.81 (m), 1.54 (m)
13	22.1, CH_3_	0.98 (d, 7.2)	11.8, CH_3_	0.90 (t, 7.2)
14	22.1, CH_3_	0.98 (d, 7.2)	17.6, CH_3_	1.21 (d, 6.6)

**Table 4:** Cytotoxic activities of compounds **1**–**10**.

Compound	IC_50_ (μM)		
	**HCT-116**	**HepG2**	**MCF-7**
**1**	30	≥50	25
**2**	NT	NT	NT
**3**	30	≥50	35
**4**	35	≥50	20
**5**	1.5	≥50	15
**6**	18	≥50	10
**7**	30	≥50	30
**8**	25	≥50	27
**9**	16	≥50	30
**10**	22	≥50	27
Doxorubicin *	0.789	0.621	0.415

* Positive control drug.
